# Blood Neurofilament Levels Predict Cognitive Decline across the Alzheimer’s Disease Continuum

**DOI:** 10.3390/ijms242417361

**Published:** 2023-12-11

**Authors:** Sylvain Lehmann, Susanna Schraen-Maschke, Jean-Sébastien Vidal, Frédéric Blanc, Claire Paquet, Bernadette Allinquant, Stéphanie Bombois, Audrey Gabelle, Constance Delaby, Olivier Hanon

**Affiliations:** 1 Laboratoire et Plateforme de Protéomique Clinique, Université de Montpellier, INM INSERM, IRMB CHU de Montpellier, 80 av Fliche, F-34295 Montpellier, France; constance.delaby@inserm.fr; 2 Univ. Lille, Inserm, CHU Lille, UMR-S-U1172, LiCEND, Lille Neuroscience & Cognition, LabEx DISTALZ, F-59000 Lille, France; susanna.schraen@inserm.fr (S.S.-M.); stephanie.bombois@aphp.fr (S.B.); 3 Université Paris Cité, INSERM U1144, GHU APHP Centre, Hopital Broca, Memory Resource and Research Centre de Paris-Broca-Ile de France, F-75013 Paris, France; jean-sebastien.vidal@aphp.fr (J.-S.V.); olivier.hanon@aphp.fr (O.H.); 4 Université de Strasbourg, Hôpitaux Universitaires de Strasbourg, Memory Resource and Research, French National Centre for Scientific Research (CNRS), ICube Laboratory UMR7357 and Fédération de Médecine Translationnelle de Strasbourg (FMTS), Team Imagerie Multimodale Intégrative en Santé (IMIS), F-67000 Strasbourg, France; f.blanc@unistra.fr; 5 Université Paris Cité, INSERM U1144, GHU APHP Nord Lariboisière Fernand Widal, Centre de Neurologie Cognitive, F-75010 Paris, France; claire.paquet@aphp.fr; 6 Université Paris Cité, Institute of Psychiatry and Neurosciences, Inserm, UMR-S 1266, F-75014 Paris, France; allinquant.bernadette@neuf.fr; 7 Assistance Publique-Hôpitaux de Paris (AP-HP), Département de Neurologie, Centre des Maladies Cognitives et Comportementales, GH Pitié-Salpêtrière, F-75013 Paris, France; 8 Université de Montpellier, CHU Montpellier, Memory Research and Resources Center, Department of Neurology, Inserm INM NeuroPEPs Team, Excellence Center of Neurodegenerative Disorders, F-34000 Montpellier, France; a-gabelle@chu-montpellier.fr; 9 Sant Pau Memory Unit, Hospital de la Santa Creu i Sant Pau, Biomedical Research Institute Sant Pau, Universitat Autònoma de Barcelona, F-08041 Barcelona, Spain

**Keywords:** Alzheimer’s disease, neurofilament light chain, blood, cognitive decline

## Abstract

Neurofilament light chain (NfL) is a potential diagnostic and prognostic plasma biomarker for numerous neurological diseases including Alzheimer’s disease (AD). In this study, we investigated the relationship between baseline plasma concentration of Nfl and Mild Cognitive Impairment in participants who did and did not have a clinically determined diagnosis of dementia by the end of the three-year study. Additionally, we explored the connection between baseline plasma concentration of NfL and AD dementia patients, considering their demographics, clinical features, and cognitive profiles. A total of 350 participants from the Biomarker of AmyLoid pepTide and AlZheimer’s diseAse Risk (BALTAZAR) multicenter prospective study were investigated: 161 AD dementia participants and 189 MCI participants (of which 141 had amnestic MCI and 48 non-amnestic MCI). Plasma biomarkers were measured at baseline and the progression of clinical and cognitive profiles was followed over the three years of follow-up. Baseline plasma NfL concentration increased across the Alzheimer’s disease continuum with a mean NfL value of 17.1 ng/mL [SD = 6.1] in non-amnestic MCI, 20.7 ng/mL [SD = 12.0] in amnestic MCI, and 23.1 ng/mL [SD = 22.7] in AD dementia patients. Plasma NfL concentration correlated with age, body mass index (BMI), and global cognitive performance and decline, as measured by the Mini-Mental State Examination (MMSE). MMSE scores decreased in parallel with increasing plasma NfL concentration, independently of age and BMI. However, NfL concentration did not predict MCI participants’ conversion to dementia within three years. Discussion: Baseline plasma NfL concentration is associated with cognitive status along the AD continuum, suggesting its usefulness as a potential informative biomarker for cognitive decline follow-up in patients.

## 1. Introduction

The population is aging and there are clear societal benefits to be gleaned from simple non-invasive tests that can track and predict the progression of cognitive decline on an individual basis [1]. Alzheimer’s disease (AD) is the most common form of dementia, and blood and cerebrospinal fluid (CSF) samples may help to identify patients at an increased risk of cognitive decline and progression to AD dementia [1]. Biomarker research in AD has also led to a shift in the way the disease is viewed as a clinico-pathophysiological entity. There is now a growing appreciation that AD should not be viewed solely as distinct, defined clinical stages, but as a multifaceted process evolving along a continuum. In the ‘Biomarker of AmyLoid pepTide and AlZheimer’s diseAse Risk’ (BALTAZAR) project, we document informative data on potential biomarkers of cognitive decline and dementia in Mild Cognitive Impairment (MCI) and AD patients [2]. Indeed, we have used CSF and blood samples from this cohort to explore the value of the constituents of neurofibrillary tangles, amyloid plaques, and neurodegeneration, i.e., phosphorylated Tau and amyloid peptides [3].

The pathophysiology of AD is associated with an accumulation of β-amyloid (Aβ) and the progressive onset of cognitive decline. The kinetics of this decline vary considerably from one individual to another, and it is therefore crucial to be able to predict clinical progression as accurately as possible for optimal treatment. Various studies have focused on the search for informative blood biomarkers of cognitive decline in this context, which have led to the identification of the Abeta ratio [3], pTau(181) [4], and pTau(217) [5], among others [6].

More recently, the neurofilament light chain (NfL) has been described as an informative and non-specific biomarker of neurodegeneration [7]. Indeed, this cytoskeletal protein, mainly present in the myelinated axons of neurons, is increased in neurodegenerative, inflammatory, vascular, or traumatic diseases [8,9]. Thus, NfL concentration rises in AD [10,11] but also in other types of dementia (such as Lewy Body Dementia or fronto-temporal dementia), and also in the context of traumatic brain injury [8]. Interestingly, its concentration in CSF and blood is well correlated so its quantification in blood remains informative of neural damage [1,8]. In this study, we explored the potential of NfL as a clinical blood marker in the clinically symptomatic AD continuum (MCI, including prodromal AD, and AD dementia). We assessed the plasma concentration of NfL in 48 non-amnestic MCI (naMCI), 141 amnestic MCI (aMCI), and 161 AD patients. Patients included in this study benefited from repeated visits and measurements of clinical and biomarker parameters to monitor the progression of the disease. Therefore, we aimed to investigate, in the whole population (MCI and AD dementia participants), the relationships between plasma NfL concentration and (i) clinical, imaging, and other plasma biomarkers, and (ii) cognitive decline over 3 years of follow-up, considering any potential confounding factors. We also investigated in the MCI participants, the relationship between plasma NfL concentration and conversion to dementia after 3 years follow-up.

## 2. Results

### 2.1. Baseline Demographic, Clinical, and Plasma Biomarker Characteristics

We analyzed 350 patients classified at baseline as AD dementia (*n* = 161), aMCI (*n* = 141), and non-amnestic MCI (naMCI, *n* = 48, Table 1). Their mean age was 76.8 (SD = 6.4) and 59% were women. AD patients were on average 15 months younger than MCI patients. The mean MMSE was significantly lower in AD patients than in naMCI patients (22.2 vs. 27.8, *p* < 0.0001). The ApoE4 carriers were more frequent in AD dementia and aMCI than in the naMCI participants (*p* = 0.017).

The hippocampal volume was lower in AD than in MCI patients (*p* < 0.001). Among the blood biomarkers tested, only plasma NfL and glycemia values were significantly different between subgroups (*p* = 0.009). There was a trend for the plasma Aβ40 and Aβ42 concentration (*p* = 0.038 and 0.018, respectively) but no significance was observed for the plasma ratio.

### 2.2. Baseline Plasma NfL Concentration and Association with Cognitive Profile

**Figure 1:**
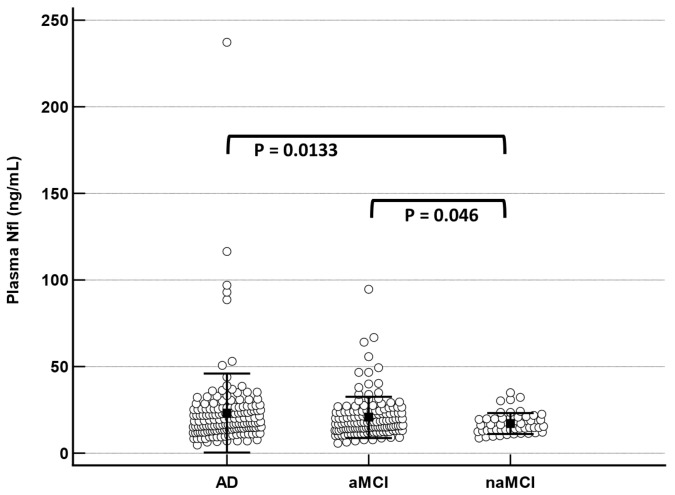
Baseline plasma NfL concentration (+/− SD) in the 350 patients as per clinical diagnosis of AD dementia, aMCI, and naMCI. NfL levels at baseline were measured in 161 AD dementia patients and 189 MCI participants, of which 141 were aMCI and 48 were naMCI. The *p*-value of the AD vs. naMCI comparison was obtained after log transformation of the data. The comparison using non-parametric Mann–Whitney test resulted in a *p*-value of 0.0289 between AD and naMCI and a *p*-value of 0.0551 between aMCI and naMCI.

First, we assessed plasma NfL concentration within the clinical groups. The plasma NfL level was statistically higher (mean 23.1 [SD = 22.7]) in AD and differed between groups (Table 1) especially between AD dementia and naMCI participants (*p* < 0.001, Figure 1 and Table 1) and between aMCI and naMCI (*p* = 0.046, Figure 1).

Next, we analyzed the relationship between NfL concentration and the global cognitive performance measures with the MMSE. There was a significant correlation between plasma NfL level and MMSE score at baseline. In addition, the CSF amyloid Aβ42/40 ratio also correlated with MMSE (Supplementary Table S1). MMSE was more strongly associated with plasma NfL concentration (*p* = 0.0005) than with CSF Aβ40/42 (*p* = 0.0236, Supplementary Table S1).

### 2.3. Plasma NfL Concentration Predicts Cognitive Decline but Not Conversion to Dementia

**Figure 2:**
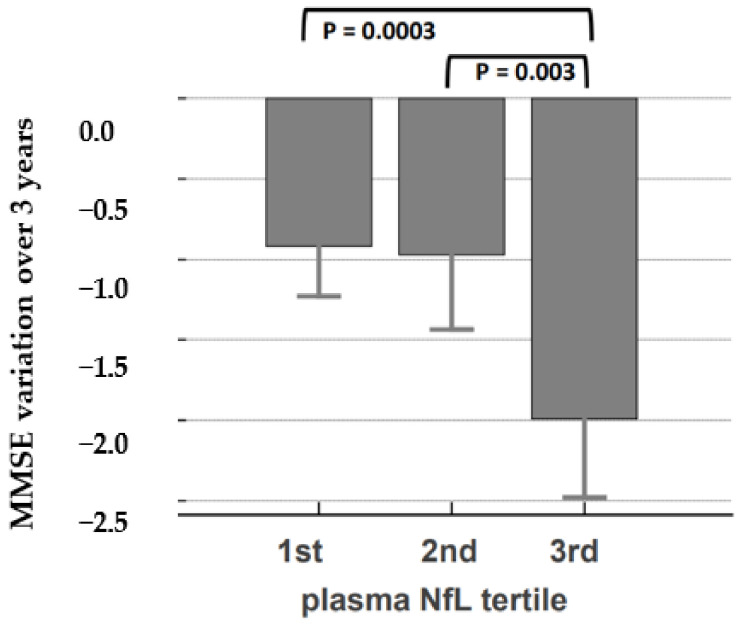
MMSE rate of variation in the different NfL tertiles. Plot showing MMSE variation over three years for the 350 participants according to plasma NfL tertile.

First, we analyzed the relevance of plasma NfL concentration in predicting global cognitive decline over time in the whole population, assessed with MMSE longitudinal data within the 3 years of follow-up. Our findings showed that the higher the plasma NfL tertile, the more cognitive performance declined over the 3 years of follow-up (Figure 2 and Table 2 and Table 3, *p* = 0.001). This contrasts with the Aβ42/40 ratio tertile, which did not have a significantly different MMSE decline (Table 3).

Then, we focused on the relevance of baseline plasma NfL concentration to identify which patients within the MCI group at baseline will convert to the dementia stage during the follow-up period. The comparison from MCI converters vs. non-converters within the 3-year follow-up highlighted a higher percentage of ApoE4 carriers, a lower baseline hippocampal volume, and lower MMSE scores in the MCI converter group (all *p*-values <0.0001, Table 2). Converters were also older, confirming that age is a risk factor for conversion. In addition, the MCI converters presented lower creatinemia levels and plasma Aβ42/40 ratios than non-converters (*p* = 0.008 and *p* = 0.01, respectively, Table 4).

When considering plasma NfL concentration in MCI patients, the higher NfL tertile had a significantly higher level of creatinemia (*p* < 0.0001), lower eGFR levels (*p* < 0.0001), and a higher level of Aβ40 (*p* = 0.0058) (Table 2). The MCI patients with the 3rd tertile were more likely to be older and have lower BMI, MMSE scores, and higher rates of cognitive decline over time (Table 2).

**Figure 3:**
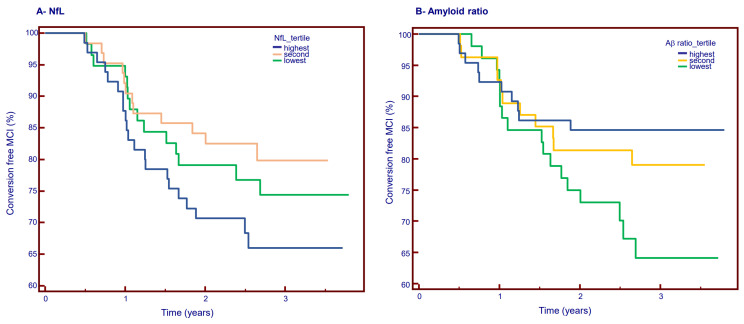
Baseline plasma NfL concentration and amyloid ratio prediction of conversion to dementia. Conversion occurrence amongst 189 MCI subjects according to tertile of (**A**) NfL and (**B**) amyloid ratio. NfL: logRank test: X^2^: 3.0068, *p* = 0.22; amyloid ratio logRank test: X^2^: 9.7546, *p* = 0.0076.

The lowest tertile of plasma Aβ42/40 significantly predicted conversion from MCI to dementia (*p* = 0.0076, Figure 3B), but this was not the case for any NfL tertile (Figure 3A). Even when the blood biomarkers Aβ1-40, Aβ1-42, Aβ1-42/Aβ1-40, and NfL were combined using a logistic regression approach, the model was not effective in detecting conversion (significance level *p* = 0.1357, resulting AUC 0.640 (95% confidence interval 0.564 to 0.712)). It is also possible that the presence of high NfL values that appear as outliners reduces the performance of the logistic regression approach. Since eGFR varies with NfL levels, it was also important to check whether its addition to the previous logistic model could improve its performance. Unfortunately, this was not the case (new significance level *p* = 0.3439, resulting AUC 0.629 (95% confidence interval 0.551 to 0.703)). The fact that the impact of renal function on blood biomarkers is non-linear, i.e., the impact is much greater at very low eGFR [4] is probably one explanation for this result.

### 2.4. Baseline Plasma NfL Concentration Is Associated with Regional Changes in Brain Volume

Grey matter and hippocampus volumes were significantly decreased and CSF volume increased in AD compared to MCI subgroups, while the Scheltens scale (assessing medial temporal lobe atrophy) increased in AD dementia (Supplementary Table S2). On the other hand, white matter volume and the Fazekas scale (measuring white matter lesions) remained stable between these subgroups. However, when considering tertiles of NfL in MCI patients, both the Scheltens and Fazekas scales increased in the highest NfL tertile compared to the lowest one (Supplementary Table S2).

Correlations were investigated among baseline plasma NfL concentration and white and grey matter volume, hippocampus volume, CSF volume, as well as Fazekas scale and Scheltens scale in the entire cohort and within each diagnostic group (Supplementary Table S3).

Baseline plasma NfL concentration significantly correlates with white matter volume in the entire cohort and MCI subgroup, while negatively correlating with grey matter volume in the entire cohort and AD dementia and MCI subgroups. The Scheltens scale was positively associated with plasma NfL concentration in these three subgroups, while the Fazekas scale only correlated with this biomarker within the entire cohort and AD dementia patients. However, there was a positive association of the Fazekas scale with the highest tertile of NfL in the MCI subgroup (Supplementary Table S3).

## 3. Discussion

Many groups have focused on the search for reliable blood biomarkers of cognitive decline in AD, leading to the identification of the Abeta ratio [3], pTau(181) [4], and pTau(217) [5,6,7,8,9,10], among others [11]. In this context, plasma NfL may be included in a biomarker panel to help detect non-AD neurodegenerative diseases (NDs) [12].

Indeed, individuals who are plasma pTau-negative but NfL-positive could be inspected for non-AD NDs, such as frontotemporal dementia, as patients who have normal values of both biomarkers are unlikely to have a progressive ND [13,14]. In general, the integration of blood NfL into a multi-marker signature is assumed to be of benefit as a screening tool to recognize patients with neurodegeneration and to better predict disease progression, as well as to monitor therapy responses in clinical trials [1,15,16].

In this paper, we have demonstrated the association of baseline plasma NfL concentration with cognitive decline within three years in AD and MCI populations. We previously demonstrated that MCI conversion to the dementia stage can be predicted by a lower plasma Aβ1-42 and Aβ1-42/Aβ1-40 ratio, independently of age, sex, education level, and APOE E4 [3]. We previously also studied the performance of plasma pTau [4]. Detection of Abeta pTau biomarkers is linked to their deposition in the brain (as described in AD, although in BALTAZAR, we do not have post-mortem information on participants).

Here, we assessed the added value of plasma NfL concentration and found that it is not a strong predictor of conversion to dementia, unlike amyloid peptides. However, the participants in the highest tertile of plasma NfL can differentiate AD dementia from amnestic MCI participants, confirming the interest of this biomarker for staging the neurodegenerative process throughout the continuum of AD pathology.

It is noteworthy that the classification of MCI into converters and non-converters is not a continuous variable but more stage-by-stage criteria, furthermore dependent on functional activities rather than on the cognitive scale. Amyloid may be more correlated to functional scores than to cognitive ones. In addition, the conversion from MCI to dementia is clearly dependent on the underlying pathology and in particular, the amyloid- and Tau-related pathology, as we specifically track the MCI due to AD that will progress to AD [17]; in the case of cognitive decline measured with the MMSE, it could be aspecific to the underlying pathology and NfL clearly appears as a marker of the neurodegenerative process but is not specific to AD. Thus, the fact that plasma baseline NfL concentration can predict disease progression, whatever the underlying pathology, is expected. Potentially, a certain percentage of patients with MCI and with dementia in BALTAZAR are not strictly AD but may include all potential etiologies of dementia.

In addition, we show that structural changes in brain regions occurring in the early clinical stage of AD are associated with plasma NfL levels, which is in agreement with previous works describing such an association with either CSF [12] or plasma NfL concentrations [18].

Therefore, whereas Aβ pathology can inform about conversion from MCI to dementia but cannot inform about cognitive decline [3], plasma NfL concentration is not predictive of conversion but is informative of MMSE decline across years of patient follow-up. The criterion of “conversion to dementia” is the result of a multimodal clinical assessment that is quite difficult to model or predict using a single marker. This is probably why NfL does not perform well in this area. The difference in MMSE score between converters and non-converters is statistically significant, but this score does not represent a biological phenomenon, and it is used to define conversion. The difference in score between the two situations is therefore expected. This conversion criterion, which is not accompanied by a clear quantitative threshold, therefore seems rather confusing, and it does not correspond to the vision of Alzheimer’s disease as a clinico-pathophysiological process evolving along a continuum. Our results are in agreement with other studies showing that plasma NfL correlates with MMSE [19] but is independent of CSF amyloid, thus suggesting its potential interest in predicting prognosis in AD patients [17,19,20]. Thus, as previously suggested, plasma NfL concentration may be of interest to predict a decline in global cognition [21,22]. Interestingly, plasma NfL was recently suggested to be a useful prognostic biomarker for PD [23], predicting clinical conversion to mild cognitive impairment or dementia [23]. As we found a significantly higher level of plasma NfL in amnestic than in non-amnestic MCI, NfL build-up could be an early event in the evolution of AD. An alternative interpretation of the fact that plasma amyloid ratio and NfL are markers for dementia conversion and cognitive decline, respectively, may be that AD is a functional impairment of neurons rather than a degradation of neurons. Clearly, to follow up on these results and test these hypotheses, it will be important to assess the correlation between baseline plasma NfL levels through other methods of assessing cognitive decline than MMSE. Of note, we also assessed ADL and IADL scores, which were differential within plasma NfL tertiles (IADL decreasing and ADL increasing in the highest tertile compared with the first, *p* < 0.05).

Future work will need a deep understanding of how AD evolves by measuring MMSE and other cognitive indicators, but also other potential confounding clinical parameters. Previous work has revealed a major impact of renal function on the plasma pTau(181) value that needs to be taken into account so as not to compromise its diagnostic performance for AD [4]. However, we previously showed that the Aβ ratio appears to be exempt from this comorbidity and remains a strong indicator of conversion to dementia and AD [5]. Here we assessed the influence of renal parameters on plasma NfL value and found a low, albeit significant, impact, Supplementary Figure S1. This is in agreement with previous work, showing an inverse correlation between plasma NfL concentration and eGFR [24]. However, the accuracy of plasma NfL in predicting conversion or cognitive decline was not affected by renal function (creatinemia or eGFR), which is in agreement with recently published data on an ADNI cohort [25].

## 4. Materials and Methods

### 4.1. Study Population

The study population corresponds to 350 participants of the BALTAZAR multicenter prospective cohort (ClinicalTrials.gov, accessed on 26 September 2023, Identifier #NCT01315639) [2] who underwent a lumbar puncture as part of the clinical protocol. All participants had clinical, neuropsychological, structural imaging, and biological assessments. APOE was genotyped in a single centralized laboratory. MCI subjects were selected according to the Petersen criteria [7]. They were dichotomized into amnestic (aMCI) and non-amnestic (naMCI) phenotypes according to the presence of memory impairment on the free and cued selective reminding test (FCSRT) (cutoff score of 40 for age ≤ 72 years and 39 for age > 72 years). Participants had visits every six months for three years. MCI participants were reassessed each time for conversion to dementia [2]. The progression from MCI to dementia was defined by evaluation of the following parameters: (i) decline in cognitive function (measured by changes from the baseline in scores of the mini-mental state examination [MMSE]), (ii) disability in activities of daily living (activities of daily living ADL > 1), and (iii) clinical dementia rating sum of boxes (>1). The conversions from MCI to dementia were reviewed by an adjudication committee and 95% converted to AD dementia, which allowed the dichotomization of MCI participants into converters and non-converters. Each participant had a physical examination performed by a physician with the calculation of body mass index (BMI). Apolipoprotein E (APOE) was genotyped in a single centralized laboratory (Centre de Biologie-Pathologie, Lille University Hospital, France) using a classical polymerase chain reaction and digestion method.

### 4.2. Ethics Approval and Consent to Participate

Written informed consent to participate in the study was provided by all participants. The BALTAZAR study has approval from the Paris ethics committee (CPP Ile de France IV Saint-Louis Hospital, Ref 2010-A00335-34).

### 4.3. Plasma and CSF Sampling and Analysis

Blood and CSF samples were collected at the same time. Investigators involved in the biological analysis were blinded to other assessments. A standard protocol was established beforehand and used throughout the study. All centers used the same 10 mL collection tube with EDTA (BD Vacutainer K2E ref 367,525; Becton Dickinson, Rungis, France). After centrifugation (2500 g, 10 min), the supernatant was aliquoted into polypropylene protein low-binding tubes (LoBind-microtube-ref 022431064; Eppendorf, Hamburg, Germany) and stored at −80 °C [26,27].

NfL concentration was determined using a commercial Simoa™ NF-light™ Advantage Kit (Quanterix™, Billerica, MA, USA) based on ultrasensitive Simoa™ HDX technology [14]. Internal quality controls (IQCs) represented by serum pool aliquots were used to monitor the accuracy of Simoa™. All samples were measured after a single thaw, with a four-fold dilution using the provided dilution buffer. Lumbar CSF samples were collected according to a standardized protocol [12,27]. High NfL values that appear as outliers are not due to an analytical problem but may correspond to patients with an exacerbation of the pathology. We therefore decided not to remove these NfL outlier values in our dataset but rather to confirm differences between groups using a non-parametric test. CSF samples (>4 mL) were centrifuged (1000 g, +4 °C, 10 min) less than 4 h after collection using the same 10 mL polypropylene tube (ref62.610.201; Sarstedt, Germany) and aliquoted into polypropylene protein low-binding tubes (LoBind microtube-ref 022431064; Eppendorf) and stored at −80 °C. CSF Aβ42 and Aβ40 levels were measured in duplicate using the same aliquot in a single centralized laboratory (IRMB, Montpellier, France) using commercially available ELISA kits (Euroimmun β-amyloid 1–40 and 1–42).

Baseline blood samples were used to measure creatinine and the estimated glomerular filtration rate (eGFR) was computed using the CKD Epidemiology Collaboration (CKD-EPI) equation, revised in 2021 without the inclusion of race [28].

### 4.4. MRI Examination

The MRI protocol included a three-dimensional volumetric T1-weighted, an axial fluid-attenuated inversion recovery T2-weighted, an axial gradient echo T2-weighted, and an axial T2-weighted fast spin echo with slices angled parallel to the axis through the genu and splenium of the corpus callosum and taken from foramen magnum to vertex. The protocol also included axial blood-oxygen-level-dependent echo planar imaging (10 min resting state) and axial diffusion tensor imaging (b-factor 5,1000 s/mm^2^; 32 directions). After MRI, the scans were sent for quality validation and postprocessing. MRI analysis was centralized and analyzed by the Centre d’acquisition et de traitement d’images [29]. Right and left hippocampal volumes were obtained for each participant using automatic segmentation of the hippocampus [30]. Hippocampal atrophy was assessed using the Scheltens scale on baseline brain MRI and the Fazekas scale was used to quantify the amount of white matter T2 hyperintense lesions.

### 4.5. Statistical Analysis

General characteristics were analyzed in the whole cohort and according to AD, aMCI, or naMCI diagnosis. Categorical variables are presented as percentage and number of subjects (% (N)) and continuous variables as mean and standard deviation (M= [SD=]), and comparisons were made with χ2 or analysis of variance. Mean levels of plasma and CSF biomarkers were calculated in the three groups and compared first with generalized linear regression with and without adjustment for age, sex, and number of APOE ε4 alleles with biomarkers as dependent variables and diagnosis treated nominally and ordinally (coded in the following order: AD, aMCI, and naMCI) to assess a linear relationship. Because all biomarkers had a skewed distribution, they were log-transformed for statistical testing, but for the sake of clarity, non-log-transformed biomarker means and standard deviations are presented. Because of the small number of naMCI patients, we also analyzed correlations in the MCI group as a whole. The results of correlations of the plasma NfL and biomarkers are presented in scatter plots with regression and 95% confidence interval lines. Statistical analysis was performed using the R statistical or Medcalc software (version 22.016). In all analyses, the two-sided a-level of 0.05 was used for significance testing.

## 5. Conclusions

Our results reveal the good performance of plasma NfL concentration in predicting cognitive decline within the AD continuum. However, this biomarker is not a very good predictor of MCI conversion to dementia. This confirms the association of NfL with neurodegenerative processes rather than with amyloid pathology, which is correlated with conversion. Plasma NfL could thus be used in clinical practice as an indicator for cognitive decline follow-up in a larger spectrum of disease stages than amyloidopathy alone.

## Figures and Tables

**Table 1:** Demographics and clinical and plasma biomarker characteristics of participants at baseline according to clinical diagnosis of AD, aMCI, and naMCI.

	All	AD	aMCI	naMCI	Anova
**Demographic characteristics**	*n* = 350	*n* = 161	*n* = 141	*n* = 48	*p*
Age (years)	76.8 (6.4)	75.9 (7.6)	77.9 (5.3)	76.4 (4.5)	**0.024**
Women (%)	208 (59)	59.6 (96)	55.3 (78)	70.8 (34)	0.167
BMI (kg/m^2^)	24.8 (3.9)	25.1 (4.4)	24.7 (3.5)	24.3 (3.6)	0.410
1 or 2 APOE4 alleles (%)	152 (43)	50.3 (78)	43.3 (61)	27.1 (13)	**0.017**
**Cognitive biomarkers**					
MMSE (/30)	24.6 (3.8)	22.2 (3.5)	26.2 (2.7) **	27.8 (1.9) **	**<0.0001**
**Brain morphological biomarkers**					
Hippocampal volume (R 1 L) (cm^3^)	4.3 (1.2)	3.9 (1.26)	4.61 (1.19) **	5.06 (0.68) **	**<0.0001**
**Blood biomarkers**					
Creatinine (μmol/L)	78.8 (22.7)	79.8 (25.2)	78.8 (21.5)	75.3 (16.9)	0.449
eGFR (mL/min/1.73 m^2^)	77.2 (15.5)	76.7 (16.1)	77.6 (15.6)	77.3 (12.8)	0.414
Plasma Aβ1-40 (ng/L)	270.3 (63.4)	261.5 (68)	279.2 (61.3)	275.5 (47.9)	**0.038**
Plasma Aβ1-42 (ng/L)	37.9 (11.5)	36.7 (11.4)	38.6 (12.1)	39.9 (10)	**0.018**
Plasma Aβ1-42/Aβ1-40 ratio	0.142 (0.04)	0.144 (0.04)	0.14 (0.04)	0.146 (0.034)	0.097
Plasma NfL (ng/L)	21.3 (17.4)	23.1 (22.7)	20.7 (12) *	17.1 (6.1) *	**0.009**
**CSF biomarkers**					
CSF Aβ1-40 (ng/L)	7262.9 (2342.4)	6601.5 (268.3)	7913.8 * (278.9)	7126.9 * (527.3)	**0.03**
CSF Aβ1-42 (ng/L)	680.8 (362.7)	556.8 (37.9)	777.6 (39.1)	792.0 (74.3)	**0.01**
CSF Aβ1-42/Aβ1-40 ratio	0.095 (0.042)	0.085 (0.004)	0.100 ** (0.004)	0.113 ** (0.008)	**<0.001**

Abbreviations: aMCI, amnestic mild cognitive impairment; ANOVA, analysis of variance; BMI, body mass index; M (SD), mean (standard deviation); MMSE, Mini-Mental State Examination; naMCI, non-amnestic mild cognitive impairment; R 1 L, right 1 left; *p*: comparison between the three groups (AD, naMCI, and aMCI) with ANOVA or χ2. Comparison between aMCI and naMCI: * *p* < 0.05, ** *p* < 0.01. Comparisons were adjusted by age, sex, and APOE4 status.

**Table 2:** Baseline characteristics in the different NfL tertiles in MCI patients.

NfL Tertiles	1st	2nd	3rd		
MCI patients per NfL tertiles	*n* = 117	*n* = 116	*n* = 117	*p*	*p*$
Age (years)	74.2 (6.1)	76.7 (6)	79.3 (6.2)	**<0.0001**	**<0.0001**
Women (%)	53.0 (62)	62.9 (73)	62.4 (73)	0.22	0.55
BMI (kg/m^2^)	25.7 (4.1)	24.8 (3.7)	24.1 (3.9)	**0.0019**	**0.0003**
MMSE at baseline (/30)	25.3 (3.3)	25.1 (3.7)	23.5 (4)	**0.0003**	**<0.0001**
MMSE variation over 3yrs	−0.9 (1.62)	−1 (2.47)	−1.96 (2.61)	**0.0005**	**0.0012**
1 or 2 APOE4 alleles (%)	45,3 (53)	44,8 (52)	40,2 (47)	0.74	0.82
Hippocampal volume (R 1 L) (cm^3^)	4.4 (1.25)	4.45 (1.12)	4.17 (1.34)	0.19	0.71
Conversion at 3 years follow up (%)	22.1 (15)	23.1 (15)	30.4 (17)	0.52	0.99
**Blood biomarkers**					
Creatinine (μmol/L)	73 (13.1)	76.8 (17.7)	86 (30.7)	**<0.0001**	**<0.0001**
eGFR (mL/min/1.73 m^2^)	83.1 (11.6)	77.9 (14.4)	70.8 (17.2)	**<0.0001**	**<0.0001**
Plasma Aβ1-40 (ng/L)	250.7 (51.8)	272.4 (56.3)	287.1 (74.3)	**<0.0001**	**0.0058**
Plasma Aβ1-42 (ng/L)	35.6 (10.4)	38 (11.7)	40 (12)	0.0046	0.21
Plasma Aβ1-42/Aβ1-40 ratio	0.145 (0.041)	0.142 (0.041)	0.142 (0.036)	0.61	0.39
Plasma NfL (ng/L)	11.6 (2.4)	17.8 (2.2)	34.2 (24.8)	**<0.0001**	**<0.0001**

Abbreviations: ANOVA, analysis of variance; BMI, body mass index; MMSE, Mini-Mental State Examination; *p*: Comparison between the three groups, with ANOVA or χ2; *p*$: comparison between the three groups with linear regression adjusted for age, sex, and the presence of the APOE ε4 allele; % (number) was used to describe categorical variables; mean ± standard deviation for continuous variables.

**Table 3:** MMSE decline according to plasma NfL and amyloid ratio tertile. MMSE decline over time in the 350 subjects is shown according to NfL and amyloid ratio tertile.

		MMSE Decline/Year			*t*-Test		
	Tertiles	1	2	3	1 vs. 2	1 vs. 3	2 vs. 3
Plasma NfL	Mean	−0.92	−0.973	−1.991	0.8518	**0.0003**	**0.0030**
	SD	1.6291	2.4495	2.6228			
Plasma Aβ1-42/Aβ1-40 ratio	Mean	−1.144	−1.593	−1.093	0.1587	0.8664	0.1115
	SD	2.2044	2.3968	2.1411			

**Table 4:** Baseline characteristics in the whole MCI population and between MCI patients who did or did not convert to dementia within the 3 years of follow-up.

	All MCI	MCI NConv	MCI Conv		
Demographic characteristics	*n* = 189	*n* = 142	*n* = 47	*p*	*p*$
Age (years)	77.5 (5.2)	76.9 (4.9)	79.3 (5.5)	**0.007**	NA
Women (%)	59.3 (112)	59.1 (84)	59.6 (28)	0.96	NA
BMI (kg/m^2^)	24.6 (3.5)	24.6 (3.5)	24.7 (3.5)	0.91	0.69
1 or 2 APOE4* alleles	60.8 (115)	31.7 (45)	61.7 (29)	**0.0002**	NA
**Cognitive biomarkers**					
MMSE (/30)	26.6 (2.6)	27.1 (2.4)	25 (2.7)	**<0.0001**	**<0.0001**
**Brain morphological biomarkers**					
Hippocampal volume (R 1 L) (cm^3^)	4.72 (1.1)	4.92 (1.01)	4.08 (1.15)	**<0.0001**	**<0.0001**
**Blood biomarkers**					
eGFR (mL/min/1.73 m^2^)	77.6 (14.9)	76.8 (15.7)	79.6 (12.2)	0.28	0.068
Plasma Aβ1-40 (ng/L)	278.2 (58)	276.8 (52.7)	283.3 (74.7)	0.54	0.80
Plasma Aβ1-42 (ng/L)	38.9 (11.6)	39.8 (11.3)	35.7 (12.1)	0.05	**0.04**
Plasma Aβ1-42/Aβ1-40 ratio	0.142 (0.039)	0.146 (0.039)	0.127 (0.032)	0.008	**0.011**
Plasma NfL (ng/L)	19.8 (10.9)	20 (11.8)	19.2 (7.4)	0.66	0.36

Abbreviations: MCI NConv, mild cognitive impairment non-converters, MCI Conv: mild cognitive impairment converters; ANOVA, analysis of variance; BMI, body mass index; mean (standard deviation); MMSE, Mini–Mental State Examination; naMCI, non-amnestic mild cognitive impairment; R 1 L, right 1 left; *p*: Comparison between the three groups, with ANOVA or χ2); *p*$: comparison between the two groups with linear regression adjusted for age, sex, and the presence of the APOE ε4 allele (* adjusted for age and sex only); % (number) was used to describe categorical variables; mean ± standard deviation for continuous variables.

## Data Availability

Data and informed consent form are available upon request after publication (APHP, Paris). Requests will be considered by each study investigator, based on the information provided by the requester, regarding the study and analysis plan. If the use is appropriate, a data sharing agreement will be put in place before distributing a fully de-identified version of the dataset, including the data dictionary used for analysis with individual participant data.

## Supplementary Materials

The supporting information can be downloaded at: https://www.mdpi.com/article/10.3390/ijms242417361/s1.
