# Supplementary material for: Blood Neurofilament Levels Predict Cognitive Decline across the Alzheimer’s Disease Continuum
Source: Int J Mol Sci. 2023 Dec 11;24(24):17361. doi: 10.3390/ijms242417361 (PMC10743700; doi:10.3390/ijms242417361)

Supplementary Table S1

Correlation table between the measured variables at baseline.

		AGE	BMI	Creatininemia	eGFR	MMSE	Plasma Aβ42/40	Plasma NfL	CSF Aβ42/40
**AGE**	Correlation Coefficient		0.058	0.066	**-0.399**	-0.064	-0.026	**0.381**	0.030
	Significance Level P		0.2796	0.2355	**<0.0001**	0.2380	0.6367	**<0.0001**	0.7135
	n		344	325	**325**	337	328	**350**	156
**BMI**	Correlation Coefficient	0.058		**0.208**	-0.166	0.033	0.112	**-0.189**	0.023
	Significance Level P	0.2796		**0.0002**	0.0029	0.5531	0.0443	**0.0004**	0.7735
	n	344		**320**	320	333	324	**344**	156
**Creatininemia**	Correlation Coefficient	0.066	**0.208**		**-0.752**	-0.049	0.049	**0.182**	-0.008
	Significance Level P	0.2355	**0.0002**		**<0.0001**	0.3906	0.3963	**0.0010**	0.9207
	n	325	**320**		**325**	314	308	**325**	151
**eGFR**	Correlation Coefficient	**-0.399**	-0.166	**-0.752**		0.077	-0.061	**-0.337**	-0.006
	Significance Level P	**<0.0001**	0.0029	**<0.0001**		0.1739	0.2826	**<0.0001**	0.9379
	n	**325**	320	**325**		314	308	**325**	151
**MMSE**	Correlation Coefficient	-0.064	0.033	-0.049	0.077		0.058	**-0.188**	0.184
	Significance Level P	0.2380	0.5531	0.3906	0.1739		0.3042	**0.0005**	0.0236
	n	337	333	314	314		318	**337**	151
**Plasma Aβ42/40**	Correlation Coefficient	-0.026	0.112	0.049	-0.061	0.058		-0.072	0.028
	Significance Level P	0.6367	0.0443	0.3963	0.2826	0.3042		0.1942	0.7333
	n	328	324	308	308	318		328	149
**Plasma NfL**	Correlation Coefficient	**0.381**	**-0.189**	**0.182**	**-0.337**	**-0.188**	-0.072		0.011
	Significance Level P	**<0.0001**	**0.0004**	**0.0010**	**<0.0001**	**0.0005**	0.1942		0.8915
	n	**350**	**344**	**325**	**325**	**337**	328		156
**CSF Aβ42/40**	Correlation Coefficient	0.030	0.023	-0.008	-0.006	0.184	0.028	0.011	
	Significance Level P	0.7135	0.7735	0.9207	0.9379	0.0236	0.7333	0.8915	
	n	156	156	151	151	151	149	156	

Spearman rank correlation coefficient: the p-values significance level was adjusted for multiple comparisons to 0.0018

Supplementary Table S2

Brain structure changes within patients at baseline.

	AD	MCI	p	MCI			p
				1st tertile NfL	2nd tertile NfL	3rd tertile NfL	
**CSF volume (mean ± SD)**	0.1972 (0.011)	0.1929 (0.012)	**0.0003**	0.1927 (0.011)	0.1941 (0.014)	0.1916 (0.010)	NS
**White matter volume (mean ± SD)**	0.3430 (0.013)	0.3417 (0.012)	NS	0.3385 (0.010)	0.3426 (0.013)	0.3433 (0.011)	NS
**Fazekas scale**	1.822 (0.696)	1.796 (0.622)	NS	1.544 (0.520)	1.956 (0.638)	1.830 (0.627)	**0.002**
**Grey matter volume (mean ± SD)**	0.459 (0.010)	0.465 (0.010)	**<0.0001**	0.4688 (0.009)	0.4633 (0.01)	0.4652 (0.08)	0.01
**Hippocampus volume (mean ± SD)**	0.0028 (0.001)	0.0034 (0.001)	**<0.0001**	0.0036 (0.0006)	0.0034 (0.0008)	0.0034 (0.0008)	NS
**Scheltens scale**	1.920 (0.920)	1.522 (0.942)	**0.0001**	1.200 (0.862)	1.525 (0.907)	1.777 (0.981)	<0.01

The P-values significance level was adjusted for multiple comparisons to 0.0083

Supplementary Table S3

Correlation table between brain structure and plasma NfL at baseline.

Correlation of plasma NfL		Entire cohort	AD	MCI (all)
**CSF volume (mm³)**	Correlation Coefficient<br>Significance Level P<br>n	0.030<br>0.6042<br>305	0.069<br>0.4141<br>143	-0.001<br>0.9853<br>162
**White matter volume (mm³)**	Correlation Coefficient<br>Significance Level P<br>n	0.140<br>0.0142<br>305	0.126<br>0.1329<br>143	0.157<br>0.0466<br>162
**Fazekas scale**	Correlation Coefficient<br>Significance Level P<br>n	**0.237**<br>**<0.0001**<br>**298**	**0.325**<br>**0.0001**<br>**138**	0.151<br>0.0571<br>160
**Grey matter volume (mm³)**	Correlation Coefficient<br>Significance Level P<br>n	**-0.211**<br>**0.0002**<br>**305**	**-0.261**<br>**0.0016**<br>**143**	-0.178<br>0.0236<br>162
**Hippocampus volume (mm³)**	Correlation Coefficient<br>Significance Level P<br>n	-0.090<br>0.1163<br>305	-0.031<br>0.7103<br>143	-0.109<br>0.1655<br>162
**Sheltens scale**	Correlation Coefficient<br>Significance Level P<br>n	**0.249**<br>**<0.0001**<br>**304**	**0.245**<br>**0.0031**<br>**144**	**0.254**<br>**0.0012**<br>**160**

Spearman rank correlation coefficient

The P-values significance level was adjusted for multiple comparisons to 0.0083

Supplementary Figure S1: Renal parameters in the three clinical groups according to plasma NfL levels. Baseline plasma NfL levels of 325 subjects are plotted against eGFR **(A)** and creatinemia (**B)** according to AD or MCI status (aMCI and naMCI). Graph shows regression line (blue) with the 95% confidence interval (red).

A **B**

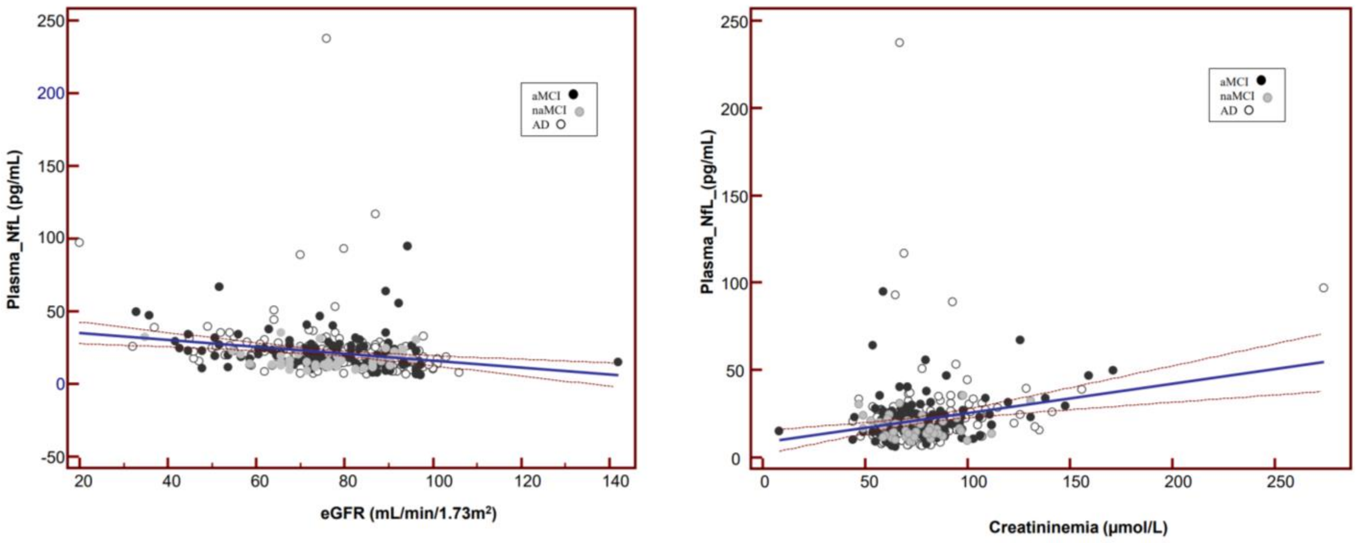

Supplement: Supplementary file 1 [file ijms-24-17361-s001.zip › ijms-2657411-supplementary.pdf]
